# Clinical Prognostic Impact of the Serum C-reactive Protein-to-albumin Ratio (CAR) in Chronic Heart Failure Patients: A Retrospective Study

**DOI:** 10.31083/j.rcm2512461

**Published:** 2024-12-25

**Authors:** Chenggong Xu, Ningli Zhang, Wei Rong, Ling Dong, Wenyi Gu, Jie Zou, Na Zhu, Tao Shi, Hao Li, Lixing Chen

**Affiliations:** ^1^Department of Cardiology, The First Affiliated Hospital of Kunming Medical University, 650032 Kunming, Yunnan, China; ^2^Department of Anesthesiology, The Second Affiliated Hospital of Kunming Medical University, 650101 Kunming, Yunnan, China; ^3^Department of Neurology, The Second Affiliated Hospital of Kunming Medical University, 650101 Kunming, Yunnan, China

**Keywords:** C-reactive protein-to-albumin ratio, C-reactive protein, heart failure with different ejection fractions, prognosis, mortality

## Abstract

**Background:**

The serum C-reactive protein-to-albumin ratio (CAR) has been identified as an adverse prognostic indicator in a variety of diseases. Nevertheless, there have been not been any studies reporting a relationship between CAR and the prognosis of chronic heart failure (CHF). This study was designed to evaluate the association between CAR and all-cause mortality in CHF patients with different ejection fractions.

**Methods:**

A total of 1221 heart failure (HF) patients were enrolled at the First Affiliated Hospital of Kunming Medical University due to acute exacerbation of chronic HF from January 2017 to October 2021. The main outcome was all-cause mortality. After collecting baseline characteristics and laboratory results from all patients, we classified all participants into four groups based on CAR quartile (G1–G4). Kaplan-Meier survival curves and multivariate Cox proportional hazard models were employed to investigate the association between CAR and all-cause mortality in the patients. Furthermore, receiver operating characteristic (ROC) curves were constructed for CARs, and the area under the curve (AUC) was calculated.

**Results:**

After excluding ineligible patients, we ultimately included 1196 patients with CHF. The mean age was 66.38 ± 12.521 years, and 62% were male. According to the Kaplan‒Meier analysis, with different ejection fractions, the risk of all-cause mortality was always highest for G4 (CAR >63.27) and lowest for G1 (CAR ≤7.67). Cox multivariate regression analyses indicated that the CAR was an independent predictor of all-cause mortality in all HF patients and in patients with different HF subtypes. According to the ROC curves, the AUC for the CAR was 0.732 (*p* < 0.001), with a sensitivity of 66.2% and the specificity of 72.7%. CAR had a greater predictive value for all-cause mortality than did C-reactive protein (CRP).

**Conclusions:**

An elevated serum CAR was independently associated with an increased risk of all-cause death, regardless of heart failure subtype.

## 1. Introduction

Heart failure (HF) comprises a group of clinical syndromic conditions characterized by pulmonary congestion and/or systemic circulation congestion with or without tissue or organ hypoperfusion, resulting in ventricular filling and/or impaired ejection capacity on account of structural and/or functional abnormalities of the heart [1]. The main clinical manifestations are dyspnoea, fatigue (limited activity tolerance) and/or fluid retention (peripheral oedema), and elevated plasma natriuretic peptide levels [2]. International guidelines based on left ventricular ejection fraction (LVEF) differences and post-treatment changes, heart failure was divided into three groups: heart failure with preserved ejection fraction (HFpEF, LVEF ≥50%), heart failure with reduced ejection fraction (HFrEF, LVEF <40%), and heart failure with mildly reduced ejection fraction (HFmrEF, LVEF 40–49%) [3, 4, 5].

Heart failure affects approximately 1–2% of the adult population in Western countries, and the total prevalence is increasing [6]. Recently, based on the medical insurance data of 50 million urban workers in China, a survey revealed the cardiac status of people ≥25 years old in China [7]. The prevalence of heart failure was approximately 1.1%, with an estimated 12.1 million people suffering from heart failure, an increase of 3 million per year [8]. These data suggest that heart failure is an enormous public health burden worldwide and that effective prevention and treatment measures are urgently needed to reduce heart failure readmissions.

Previous studies have shown an association between a person’s nutritional status and levels of inflammation which are often accompanied by adverse outcomes or a risk of death [9, 10]. Many biomarkers are associated with the development, progression, and outcome of heart failure. Elevated inflammatory factor levels and poor nutritional status are hallmarks of advanced heart failure; they are common problems in hospitalized HF patients and are associated with adverse outcomes [11].

C-reactive protein (CRP) is a trace protein found in the circulating blood of healthy people. When the body experiences inflammation and infection, CRP levels increase [12, 13, 14]. The primary source of CRP is liver cells, which are induced by cytokines like interleukin-6 (IL-6) [15]. Over the course of heart failure, a decrease in cardiac output, myocardial hypoxia, and myocardial damage can trigger an upsurge in IL-6 production, which in turn raises CRP levels. This can subsequently stimulate the generation of complement and cytokines within the immune system by CRP, exacerbating inflammation and contributing to the worsening of heart failure, ultimately leading to a poor prognosis [16, 17]. CRP serves as a crucial indicator not only for acute inflammation but also for chronic inflammation. CRP has been demonstrated to be a poor prognostic indicator for coronary artery disease and a significant risk factor for cardiovascular disease [14]. At the same time, albumin is the most important and common protein in human plasma. It is synthesized in the liver and is an indispensable nutrient for the human body. It can maintain the stability of plasma osmotic pressure, and can be combined with a variety of nutrients, hormones and drugs, accounting for about 50% of the total plasma protein. It can indicate the body’s nutritional status, and can also detect diseases that affect the metabolic function of the liver [18]. When albumin is reduced, it indicates that there are some diseases in the body, such as malnutrition, inflammation, liver diseases, tumors and so on. Patients suffering from heart failure often have low serum albumin due to inflammation and malnutrition. A previous study has mostly considered albumin decline as a risk factor for patients suffering from heart failure, and patients suffering from heart failure complicated with hypoproteinemia usually have a poor prognosis [19].

In clinical practice, CRP levels usually indicate the extent of inflammation, whereas the serum concentration of albumin (ALB) can serve as a nutritional indicator in individuals with severe health issues. Both serum CRP and ALB are valuable prognostic manifests for determining the mortality risk of patients suffering from HF [13, 14]. In clinical practice, the ratio of serum CAR can be utilized for assess whether patients suffering from heart failure have a combination of inflammation and malnutrition. In prior studies, it was considered an independent prognostic marker for patients with malignancy, infection, haemodialysis, or critical illness [20, 21, 22, 23, 24, 25, 26]. Recent studies by Kalyoncuoglu M and Durmus G [22] have shown that CAR is an effective marker for predicting coronary heart disease. According to these findings, we believe that the serum CAR can serve as a predictive indicator for the risk of mortality in patients diagnosed with CHF. Therefore, we need to further discuss whether the novel inflammatory marker CAR can be used for prognostic evaluation in patients with CHF [21, 22, 23].

## 2. Methods

### 2.1 Study Population

Through the electronic medical record system of the First Affiliated Hospital of Kunming Medical University, we inquired about patients with heart failure who were hospitalized from January 2017 to October 2021. Finally, we enrolled 1221 patients with an acute exacerbation of CHF. Our study included patients with HF who were categorized as New York Heart Association (NYHA) functional Class III or IV based on signs and symptoms. It also includes an increase in the level of natriuretic peptide, which is mainly a rapid increase in the level of B-type natriuretic peptide (BNP) or N-terminal pro-BNP (NT-proBNP), usually BNP >100 ng/L, NT-proBNP >300 ng/L. We enrolled 1196 patients with heart failure in the study after excluding individuals with missing data (such as CRP, ALB, or lgBNP), those with other significant medical conditions (such as cancer, severe infection: use anti-infective drugs multiple times, neoplastic hematologic disorder, severe kidney disease: patients already on regular dialysis or with GFR <15 mL/min, severe liver disease: patients diagnosed with cirrhosis or liver failure).

### 2.2 Data Collection

At the time of admission, data including demographic and clinical information, electrocardiograms, cardiac ultrasound results, and blood samples were gathered. Data on patient age and sex were also collected. Clinical information included heart rate (HR), blood pressure (BP), body mass index (BMI), NYHA cardiac function classification, medical history, treatment history, and left ventricular ejection fraction (LVEF). Blood tests included BNP levels, white blood cell (WBC) counts, red blood cell (RBC) counts, neutrophil (NBC) counts, lymphocyte (LBC) counts, serum CRP levels, serum haemoglobin (Hb) levels, platelet (PLT) counts, serum sodium, serum potassium, serum chlorine, serum albumin (ALB), alanine aminotransferase (ALT), aspartate aminotransferase (AST), serum creatinine (Cre), serum uric acid (UA), total cholesterol (TC) and glucose. To minimize food and drug interference with the results, blood samples were collected prior to medication and other treatments. Blood samples were collected from all patients after an overnight fasting period of 8 to 12 hours and then transported to the laboratory.

The following formula was utilized to calculate the CAR: CAR = C-reactive protein/albumin × 100.

Researchers gathered information on survival by interviewing patients or their relatives over the phone. If there was no response, the follow-up concluded with the patient’s latest medical records. The main focus of the study was to determine all-cause mortality.

### 2.3 Ethics

Prior to the commencement of the study, all participating patients duly completed and signed informed consent forms. The ethical principles outlined in the Declaration of Helsinki were rigorously adhered to throughout the course of the research. Furthermore, the research project received approval from the Human Ethics Committee of the First Affiliated Hospital of Kunming Medical University (application ID: (2022) Ethics L No. 173).

### 2.4 Statistical Analysis

According to the quartiles of CAR, the patients in the study were categorized into four groups: Group 1—CAR ≤7.67 (n = 299), Group 2—7.67 < CAR ≤ 20.95 (n = 299); Group 3—20.95 < CAR ≤ 63.27 (n = 299); and Group 4—CAR >63.27 (n = 299). When describing patient baseline characteristics, for continuous variables that follow a normal distribution, the mean and standard deviation are utilized. Conversely, for continuous variables that do not conform to a normal distribution, the median and interquartile range are employed. The results of categorical variables are expressed in terms of frequency and percentage. To compare baseline characteristics among the four groups, variance analyses were utilized for continuous variables with a normal distribution, Mann‒Whitney U tests for non-normally distributed data, and Chi-square tests for categorical variables. Survival duration was analyzed through Kaplan-Meier curves, and differences in survival probabilities between groups were assessed using the log-rank test. Cox proportional hazards models were utilized to investigate the relationship between CAR and mortality. The study utilized univariate Cox proportional hazard regression analysis to evaluate the impact of individual variables on all-cause mortality. Subsequently, multivariate Cox proportional hazard regression analysis was conducted for variables with p values less than 0.05 in the univariate analysis to identify independent predictors of all-cause mortality in patients with heart failure. Receiver operating characteristic (ROC) analysis was employed to assess the predictive value of CRP combined with ALB for all-cause mortality in HF patients. All statistical analyses were performed using SPSS version 26.0 (SPSS, Inc., Chicago, IL, USA). A *p*-value of less than 0.05 was deemed to indicate statistical significance.

## 3. Results

### 3.1 Baseline Patient Characteristics

**Fig. 1.**
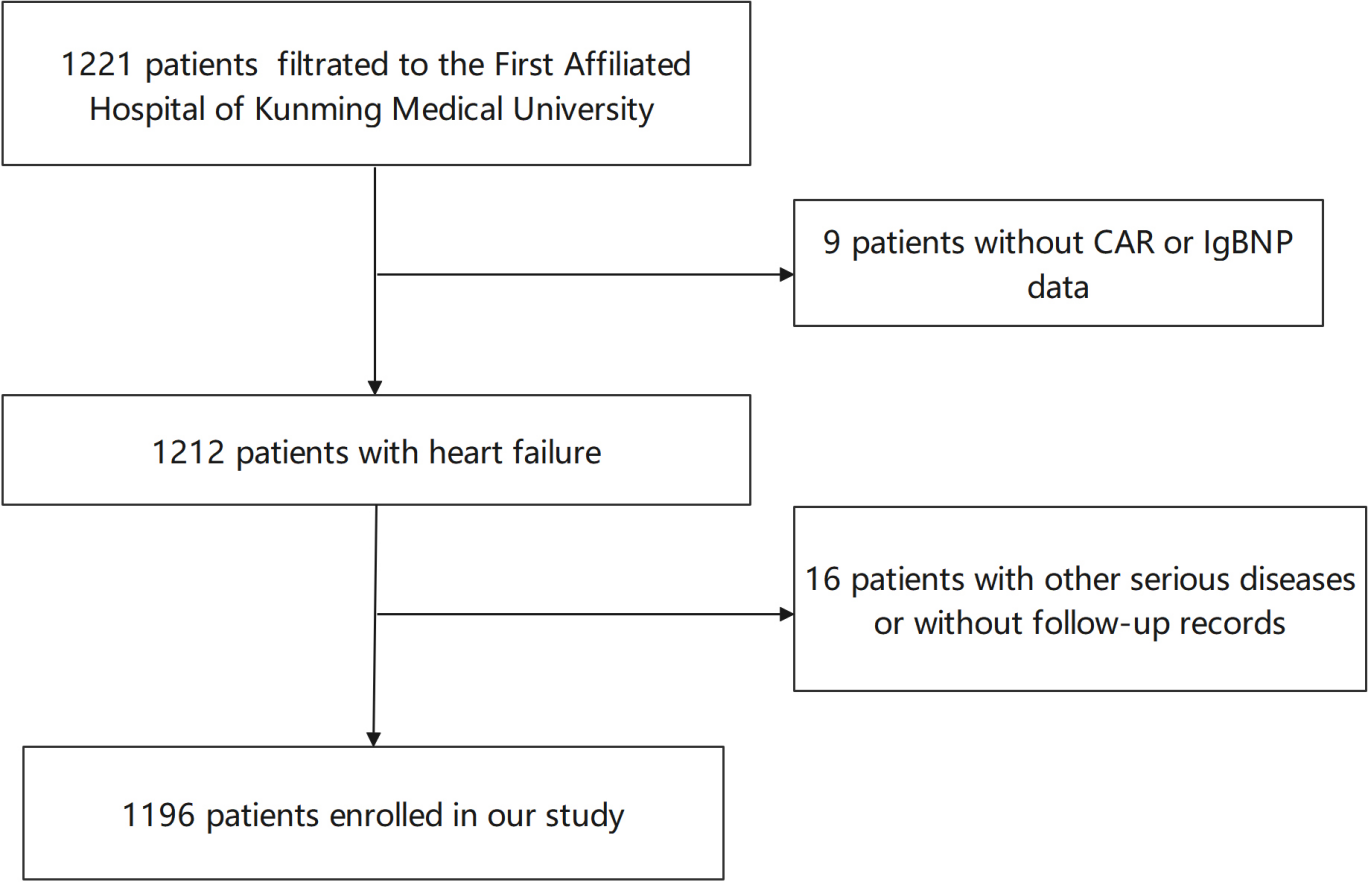
**Flow-chart of study patients**. CAR, c-reactive protein-to-albumin ratio; BNP, brain natriuretic peptide.

Patients with lost follow-up and missing data were excluded, meaning 1196 patients with CHF were analysed (Fig. 1). Overall, the mean age was 66.38 ± 12.521 years, and 62% of the patients were male (*p* < 0.0001). Based on the CAR quartile results, we have categorized all patients into four groups: Group 1 (n = 299), Group 2 (n = 299), Group 3 (n = 299) and Group 4 (n = 299). We identified statistically significant differences among the four groups in age, heart rate, coronary heart disease, CRP, ALB, lg BNP, AST, creatinine, uric acid, glomerular filtration rate, glucose, total cholesterol, high density lipoprotein cholesterol (HDL-C), low density lipoprotein cholesterol (LDL-C), potassium, sodium, chlorine, WBC, neutrophil, lymphocyte, RBC, haemoglobin, fibrinogen, NYHA cardiac function classification (class IV), angiotensin converting enzyme inhibitor (ACEI)/angiotensin II receptor blocker (ARB)/angiotensin receptor-enkephalinase inhibitor (ARNI), Beta blockers, Diuretics, Aldosterone antagonist (*p* < 0.05) (Table 1).

**Table 1.** **Baseline characteristics according to CAR**.

Variables			C-reactive protein to albumin ×100				
		(n = 1196)	G1 (n = 299)	G2 (n = 299)	G3 (n = 299)	G4 (n = 299)	*p*
Demographic data							
	Age, (years)	66.38 ± 12.521	63.91 ± 11.967	66.86 ± 12.919	68.03 ± 12.349	68.52 ± 12.378	<0.001
	Male (%)	742 (62)	173 (57.9)	179 (59.9)	198 (66.2)	192 (64.2)	0.131
	Body mass index, (kg/m^2^)	23.02 ± 3.81	23.09 ± 3.79	23.03 ± 4.13	23.03 ± 3.65	22.93 ± 3.67	0.963
	Heart rate, (beat/minute)	82 (70, 97)	79 (67, 93)	82 (70, 97)	81 (70, 96)	89 (75, 105)	<0.001
	Systolic blood pressure, (mmHg)	122.10 ± 22.94	120.93 ± 22.29	124.07 ± 22.52	122.80 ± 23.18	120.59 ± 23.69	0.206
	Diastolic blood pressure, (mmHg)	76.23 ± 15.04	76.41 ± 15.08	76.84 ± 15.43	76.39 ± 14.42	75.29 ± 15.25	0.631
	LVEF	45.48 ± 16.516	45.05 ± 17.129	46.5 ± 15.627	44.10 ± 16.390	46.19 ± 16.844	0.245
	Hypertension (%)	660 (55.2)	158 (52.8)	163 (54.5)	173 (57.9)	166 (55.5)	0.660
	Diabetes mellitus (%)	341 (28.5)	70 (23.4)	84 (28.1)	97 (32.4)	90 (30.1)	0.091
	Coronary heart disease (%)	619 (51.8)	136 (45.5)	169 (56.5)	150 (50.2)	164 (54.8)	0.031
	History of stroke (%)	167 (14.0)	36 (12.0)	35 (11.7)	52 (17.4)	44 (14.7)	0.154
	Atrial fibrillation (%)	406 (33.9)	95 (31.8)	111 (37.1)	110 (36.8)	90 (30.1)	0.170
	Smoking status (%)	409 (34.2)	96 (32.1)	94 (31.4)	102 (34.1)	117 (39.1)	0.185
	Drinking status (%)	201 (16.8)	56 (18.7)	44 (14.7)	48 (16.1)	53 (17.7)	0.567
Laboratory data							
	CRP, (mg/L)	7.47 (3.00, 21.78)	1.60 (0.76, 2.30)	5.00 (3.50, 6.10)	12.9 (10.5, 16.52)	47.88 (29.3, 89.97)	<0.001
	Albumin, (g/dL)	36.6 (34.0, 39.6)	38.4 (35.3, 41.7)	37.3 (34.9, 39.5)	36 (33.8, 39)	34.7 (31.7, 37.4)	<0.001
	LgBNP	3.17 ± 0.28	3.11 ± 0.26	3.15 ± 0.27	3.19 ± 0.28	3.23 ± 0.30	<0.001
	ALT, (IU/L)	25.05 (16.7, 42.3)	24.3 (16.4, 38.4)	24.2 (16.9, 41.0)	25.3 (16.7, 41.4)	28.3 (16.4, 57.7)	0.066
	AST, (IU/L)	28.6 (20.03, 43.28)	26.8 (20, 36.9)	28.5 (20.3, 40.7)	29.6 (19.9, 45)	30 (21, 66)	0.003
	Creatinine, (µmol/L)	103.5 (83.2, 134.1)	96.5 (79.1, 127.8)	100.9 (83.0, 124.7)	106.5 (86.3, 134.2)	112.1 (85.5, 151.87)	<0.001
	Uric acid, (µmol/L)	477.1 (370.5, 588.1)	461.5 (361, 563.8)	462.0 (369.3, 506.4)	499.5 (394.85, 611.58)	497.85 (370.38, 614.73)	0.004
	Glomerular filtration rate, (mL/min)	44.1 (32.33, 56.69)	47.74 (35.49, 61.08)	44.56 (34.80, 56.25)	43.33 (31.64, 54.16)	40.57 (26.71, 54.78)	<0.001
	Glucose, (mmol/L)	5.03 (4.16, 6.5)	4.93 (4.13, 5.8)	4.93 (4.12, 6.20)	5.1 (4.16, 6.8)	5.12 (4.23, 7.4)	0.009
	Triglyceride, (mmol/L)	1.27 ± 0.71	1.29 ± 0.67	1.28 ± 0.65	1.26 ± 0.84	1.26 ± 0.68	0.938
	Total cholesterol, (mmol/L)	3.64 ± 1.02	3.8 ± 51.02	3.69 ± 0.97	3.61 ± 0.98	3.42 ± 1.05	<0.001
	HDL-C, (mmol/L)	0.99 ± 0.32	1.08 ± 0.30	1.01 ± 0.31	0.98 ± 0.32	0.90 ± 0.32	<0.001
	LDL-C, (mmol/L)	2.30 ± 0.88	2.42 ± 0.86	2.30 ± 0.85	2.24 ± 0.86	2.23 ± 0.92	0.031
	Potassium, (mmol/L)	3.9 (3.55, 4.27)	3.9 (3.62, 4.20)	3.91 (3.61, 4.32)	3.86 (3.47, 4.22)	3.9 (3.54, 4.33)	0.049
	Sodium, (mmol/L)	141.4 (138.4, 143.9)	141.8 (138.9, 144.5)	141.8 (139.1, 144.1)	141.4 (138.5, 143.9)	140.2 (136.7, 142.9)	<0.001
	Chlorine, (mmol/L)	102.92 ± 4.68	103.91 ± 4.43	103.35 ± 4.46	102.64 ± 4.50	101.78 ± 5.05	<0.001
	WBC, (10^9^/L)	6.93 (5.54, 9.07)	6.29 (5.27, 8.02)	6.54 (5.40, 8.23)	7.18 (5.74, 9.03)	8.13 (6.05, 10.96)	<0.001
	Neutrophil, (10^9^/L)	4.53 (3.49, 6.50)	4.03 (3.14, 5.5)	4.17 (3.33, 5.61)	4.83 (36.8, 6.36)	5.75 (4.01, 8.75)	<0.001
	Lymphocyte, (10^9^/L)	1.50 ± 0.76	1.63 ± 0.74	1.56 ± 0.88	1.48 ± 0.70	1.31 ± 0.66	<0.001
	RBC, (10^12^/L)	4.54 ± 0.77	4.62 ± 0.72	4.54 ± 0.72	4.63 ± 0.79	4.39 ± 0.81	<0.001
	Hemoglobin, (g/L)	140 (124, 154)	142 (127, 155)	139 (125, 152)	142 (126, 156)	135 (117, 149)	<0.001
	Platelet, (10^9^/L)	200.64 ± 78.65	205.62 ± 75.41	198.85 ± 75.55	198.30 ± 79.92	199.79 ± 83.60	0.648
	Fib, (g/L)	3.37 (2.71, 4.15)	3.02 (2.56, 3.72)	3.19 (2.67, 3.89)	3.54 (2.85, 4.14)	4 (2.88, 5.08)	<0.001
NYHA cardiac function classification, *n* (%)							
	Class IV	444 (37.1)	79 (26.4)	110 (36.8)	124 (41.5)	131 (43.8)	<0.001
Treatment, n (%)							
	ACEI or ARB or ARNI	415 (34.7%)	73 (24.4%)	96 (32.1%)	129 (43.1%)	117 (39.1%)	<0.001
	Beta blockers	649 (54.3%)	128 (42.8%)	147 (49.2%)	179 (59.9%)	195 (65.2%)	<0.001
	Diuretics	968 (80.9%)	195 (65.2%)	238 (79.6%)	256 (85.6%)	279 (93.3%)	<0.001
	Aldosterone antagonist	811 (67.8%)	164 (54.8%)	185 (61.9%)	217 (72.6%)	245 (81.9%)	<0.001
	CRT/CRTD	116 (9.7%)	22 (7.4%)	26 (8.7%)	35 (11.7%)	33 (11.0%)	0.241

Differences in normally distributed continuous variables were compared using variance analyses, and those in nonnormally distributed data were compared using Mann‒Whitney U tests. Chi-square tests were used to compare differences in categorical variables among 4 groups. The *p*-value is obtained by comparing the groups. *p* < 0.05 was considered indicative of statistical significance. CAR, c-reactive protein-to-albumin ratio; CRP, c-reactive protein; BNP, brain natriuretic peptide; ALT, alanine transaminase; AST, aspartate transaminate; HDL-C, high density lipoprotein cholesterol; LDL-C, low density lipoprotein cholesterol; WBC, white blood cells; RBC, red blood cells; Fib, fibrinogen; NYHA, New York Heart Association; ACEI, angiotensin converting enzyme inhibitor; ARB, angiotensin II receptor blocker; ARNI, angiotensin receptor-enkephalinase inhibitor; CRT, cardiac resynchronisation therapy; CRTD, cardiac resynchronisation therapy defbrillator; LVEF, left ventricular ejection fraction. G1: CAR ≤7.67, G2: 7.67 < CAR ≤ 20.95, G3: 20.95 < CAR ≤ 63.27, G4: CAR >63.27.

### 3.2 Associations between the CAR and All-Cause Mortality

We conducted Kaplan-Meier analysis to investigate the association between CAR and all-cause mortality in patients diagnosed with heart failure (Fig. 2A, Fig. 2B, Fig. 2C). Among heart failure patients exhibiting varying ejection fractions, the Kaplan-Meier analysis revealed that patients in Group 1 (CAR ≤7.67) with heart failure exhibited the lowest cumulative incidence of all-cause mortality, lower in Group 2 (7.67 < CAR ≤ 20.95) and increased gradually in Group 3 (20.95 < CAR ≤ 63.27) and Group 4 (CAR >63.27), all patients—log-rank χ^2^ 193.396, *p* < 0.001; HFpEF patients—log-rank χ^2^ 116.530, *p* < 0.001; HFrEF patients plus HFmrEF patients—log-rank χ^2^ 126.205, *p* < 0.001.

**Fig. 2A.**
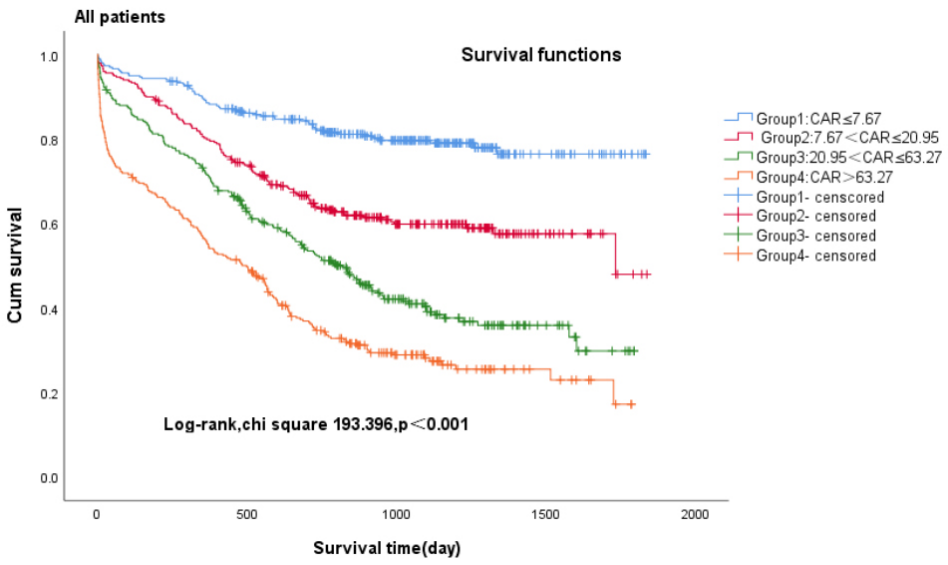
**Kaplan-Meier survival curves for all patients with CHF across CAR quartiles**. Group 1 (CAR ≤7.67), Group 2 (7.67 < CAR ≤ 20.95), Group 3 (20.95 < CAR ≤ 63.27), Group 4 (CAR >63.27). CHF, chronic heart failure; CAR, c-reactive protein-to-albumin ratio.

**Fig. 2B.**
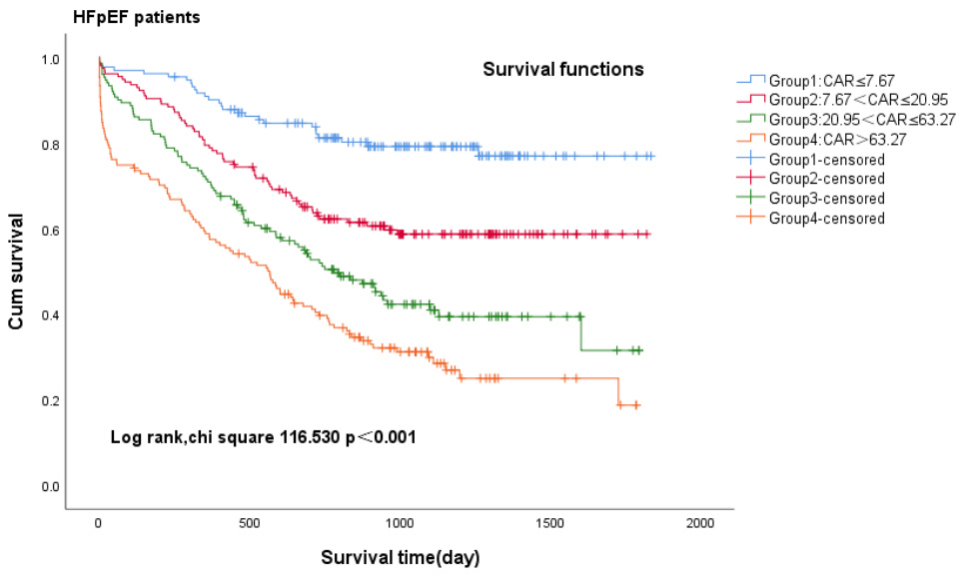
**Kaplan-Meier survival curves for HFpEF patients with CHF across CAR quartiles**. Group 1 (CAR ≤7.67), Group 2 (7.67 < CAR ≤ 20.95), Group 3 (20.95 < CAR ≤ 63.27), Group 4 (CAR >63.27). CAR, c-reactive protein-to-albumin ratio; HFpEF, heart failure with preserved ejection fraction; CHF, chronic heart failure.

**Fig. 2C.**
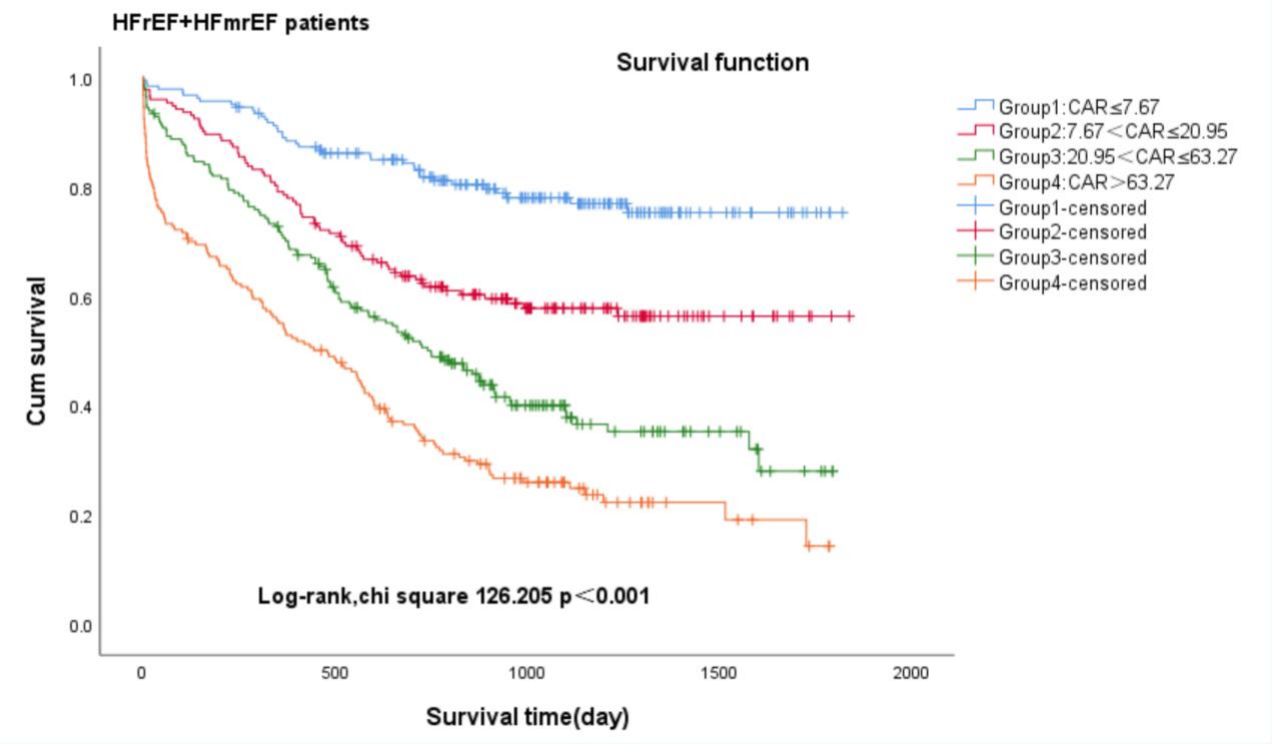
**Kaplan-Meier survival curves for HFrEF plus HFmrEF patients with CHF across CAR quartiles**. Group 1 (CAR ≤7.67); Group 2 (7.67 < CAR ≤ 20.95); Group 3 (20.95 < CAR ≤ 63.27); Group 4 (CAR >63.27). CAR, c-reactive protein-to-albumin ratio; HFrEF, heart failure with reduced ejection fraction; HFmrEF heart failure with mildly reduced ejection fraction; CHF, chronic heart failure.

### 3.3 CAR as a Predictor of Adverse Outcomes

After correcting for age, sex, NYHA class, LVEF, heart rate, SBP, DBP, CRP, ALB, ALT, AST, TC, creatinine, uric acid, chlorine, WBC, haemoglobin and lg BNP, multivariate analysis was performed. Based on the results of multivariate Cox proportional hazard analysis, it is suggested that CAR may serve as an independent predictor of all-cause mortality in patients with heart failure. All patients with heart failure (CAR: HR 1.006, 95% CI 1.002, 1.009; *p* = 0.019), HFpEF heart failure (CAR: HR 1.005, 95% CI 1.002, 1.0009, *p* = 0.028), and HErEF plus HEmrEF heart failure (CAR: HR 1.007, 95% CI 1.001, 1.010, *p* = 0.019) were included (Table 2A, Table 2B, Table 2C). Based on the CAR quartile grouping, we calculated hazard ratios for mortality in patients with HF. Among heart failure patients with different ejection fractions, patients in Group 4 always had the highest risk of death when Group 1 was used as a reference. According to Model 3, among all the HF patients, the risk of death in Group 4 was 2.529 times greater than that in Group 1; among the HFpEF patients, the risk was 3.087 times greater; and among the HFrEF plus HFmrEF patients, the risk was 3.827 times greater (*p* < 0.05) (Table 3).

**Table 2A.** **Univariable and multivariable cox proportional hazard models for serum CAR in all heart failure**.

	Univariable		Multivariable	
	HR (95% CI)	*p*	HR (95% CI)	*p*
Age	1.030 (1.023, 1.038)	<0.001	1.024 (1.016, 1.033)	<0.001
Sex (reference: male)	1.037 (0.874, 1.230)	0.680	0.991 (0.823, 1.193)	
NYHA class (reference: class IV)	0.412 (0.349, 0.486)	<0.001	0.491 (0.412, 0.586)	<0.001
LVEF	0.994 (0.989, 1.000)	0.033	1.000 (0.993, 1.006)	
Heart rate	1.007 (1.003, 1.010)	<0.001	1.005 (1.001, 1.009)	0.020
SBP	0.994 (0.990, 0.998)	0.001	0.999 (0.993, 1.004)	
DBP	0.985 (0.980, 0.991)	<0.001	0.992 (0.984, 1.000)	
CRP	1.011 (1.009, 1.012)	<0.001	1.013 (1.010, 1.018)	<0.001
Albumin	0.931 (0.913, 0.949)	<0.001	0.956 (0.934, 0.980)	<0.001
ALT	1.003 (1.002, 1.004)	<0.001	1.000 (0.998, 1.001)	
AST	1.004 (1.003, 1.005)	<0.001	1.003 (1.001, 1.005)	0.004
TC	0.818 (0.749, 0.893)	<0.001	1.012 (0.927, 1.105)	
Creatinine	1.003 (1.002, 1.003)	<0.001	1.000 (0.984, 1.001)	
Uric acid	1.001 (1.000, 1.002)	<0.001	1.002 (1.001, 1.003)	<0.001
Chlorine	0.930 (0.914, 0.947)	<0.001	0.956 (0.939, 0.974)	<0.001
WBC	1.051 (1.030, 1.071)	<0.001	1.057 (1.035, 1.061)	
Hemoglobin	0.990 (0.987, 0.994)	<0.001	0.994 (0.990, 0.998)	0.002
LgBNP	5.291 (3.888, 7.200)	<0.001	2.815 (1.949, 4.066)	<0.001
CAR	1.004 (1.003, 1.005)	<0.001	1.006 (1.002, 1.009)	0.019

Corrected for age, NYHA class, heart rate, SBP, DBP, CRP, albumin, ALT, AST, TC, creatinine, uric acid, chlorine, WBC, hemoglobin, lgBNP. HR, hazard ratio; CI, confidence interval; CAR, c-reactive protein-to-albumin ratio; NYHA, New York Heart Association; LVEF, left ventricular ejection fraction; SBP, systolic blood pressure; DBP, diastolic blood pressure; CRP, c-reactive protein; ALT, alanine transaminase; AST, aspartate transaminsae; TC, total cholesterol; WBC, white blood cells; BNP, brain natriuretic peptide.

**Table 2B.** **Univariable and multivariable cox proportional hazard models for serum CAR in HFpEF heart failure**.

	Univariable		Multivariable	
	HR (95% CI)	*p*	HR (95% CI)	*p*
Age	1.045 (1.031, 1.060)	<0.001	1.036 (1.020, 1.052)	<0.001
Sex (reference: male)	0.890 (0.672, 1.180)	0.419	0.892 (0.649, 1.221)	
NYHA class (reference: class IV)	0.377 (0.286, 0.498)	<0.001	0.397 (0.290, 0.544)	<0.001
Heart rate	1.005 (1.001, 1.012)	<0.001	1.006 (1.005, 1.011)	
SBP	0.997 (0.991, 1.003)	0.315	0.998 (0.995, 1.006)	
DBP	0.987 (0.978, 0.996)	0.005	0.997 (0.985, 1.010)	
CRP	1.011 (1.009, 1.012)	<0.001	1.015 (1.008, 1.021)	0.027
Albumin	0.919 (0.892, 0.947)	<0.001	0.956 (0.934, 0.980)	0.031
ALT	1.004 (1.002, 1.007)	<0.001	0.097 (0.993, 1.001)	
AST	1.007 (1.005, 1.009)	<0.001	1.009 (1.005, 1.013)	<0.001
TC	0.826 (0.804, 0.897)	<0.001	1.096 (0.922, 1.292)	
Creatinine	1.002 (1.001, 1.003)	<0.001	1.001 (0.998, 1.003)	
Uric acid	1.001 (1.000, 1.002)	0.035	1.002 (1.001, 1.004)	<0.001
Chlorine	0.924 (0.899, 0.950)	<0.001	0.932 (0.905, 0.971)	<0.001
WBC	1.042 (1.009, 1.077)	0.012	0.960 (0.922, 1.000)	0.049
Hemoglobin	0.989 (0.983, 0.994)	<0.001	0.994 (0.987, 0.999)	0.043
LgBNP	6.086 (3.659, 10.123)	<0.001	3.355 (1.849, 6.086)	<0.001
CAR	1.002 (1.003, 1.004)	<0.001	1.005 (1.002, 1.009)	0.028

Corrected for age, NYHA class, heart rate, SBP, DBP, CRP, albumin, ALT, AST, TC, creatinine, uric acid, chlorine, WBC, hemoglobin, lgBNP. HR, hazard ratio; CI, confidence interval; CAR, c-reactive protein-to-albumin ratio; NYHA, New York Heart Association; SBP, systolic blood pressure; DBP, diastolic blood pressure; CRP, c-reactive protein; ALT, alanine transaminase; AST, aspartate transaminsae; TC, total cholesterol; WBC, white blood cells; BNP, brain natriuretic peptide; HFpEF, heart failure with preserved ejection fraction.

**Table 2C.** **Univariable and multivariable cox proportional hazard models for serum CAR in HFrEF plus HFmrEF heart failure**.

	Univariable		Multivariable	
	HR (95% CI)	*p*	HR (95% CI)	*p*
Age	1.027 (1.018, 1.036)	<0.001	1.022 (1.011, 1.032)	<0.001
Sex (reference: male)	1.041 (0.839, 1.291)	0.717	1.049 (0.846, 1.386)	
NYHA class (reference: class IV)	0.438 (0.356, 0.538)	<0.001	0.547 (0.436, 0.685)	<0.001
Heart rate	1.005 (1.008, 1.013)	0.015	1.004 (1.001, 1.007)	
SBP	0.997 (0.991, 0.997)	0.001	1.001 (0.993, 1.009)	
DBP	0.984 (0.977, 0.991)	<0.001	0.989 (0.978, 1.010)	
CRP	1.010 (1.008, 1.013)	<0.001	1.012 (1.005, 1.019)	0.031
Albumin	0.935 (0.912, 0.959)	<0.001	0.952 (0.920, 0.985)	0.005
ALT	1.003 (1.002, 1.004)	<0.001	1.001 (1.000, 1.003)	
AST	1.004 (1.002, 1.005)	<0.001	1.004 (1.002, 1.009)	0.046
TC	0.811 (0.729, 0.903)	<0.001	0.927 (0.896, 0.997)	
Creatinine	1.004 (1.003, 1.005)	<0.001	1.001 (0.998, 1.002)	
Uric acid	1.002 (1.000, 1.003)	<0.001	1.001 (1.000, 1.005)	0.001
Chlorine	0.936 (0.914, 0.958)	<0.001	0.972 (0.949, 0.997)	0.025
WBC	1.057 (1.032, 1.083)	0.012	0.998 (0.945, 1.004)	
Hemoglobin	0.991 (0.986, 0.995)	<0.001	0.994 (0.989, 0.998)	0.021
LgBNP	6.073 (3.965, 9.302)	<0.001	2.695 (1.649, 4.286)	<0.001
CAR	1.004 (1.003, 1.005)	<0.001	1.007 (1.001, 1.010)	0.019

Corrected for age, NYHA class, heart rate, SBP, DBP, CRP, albumin, ALT, AST, TC, creatinine, uric acid, chlorine, WBC, hemoglobin, lgBNP. HR, hazard ratio; CI, confidence interval; CAR, c-reactive protein-to-albumin ratio; NYHA, New York Heart Association; SBP, systolic blood pressure; DBP, diastolic blood pressure; CRP, c-reactive protein; ALT, alanine transaminase; AST, aspartate transaminsae; TC, total cholesterol; WBC, white blood cells; BNP, brain natriuretic peptide; HFrEF, heart failure with reduced ejection fraction; HFmrEF, heart failure with mildly reduced ejection fraction.

**Table 3.** **Hazard ratios for mortality of patients with heart failure according to the quartile of CAR**.

	Unadjusted		Model 1		Model 2		Model 3	
	HR (95% CI)	*p*	HR (95% CI)	*p*	HR (95% CI)	*p*	HR (95% CI)	*p*
All								
G1	1		1		1		1	
G2	2.198 (1.601, 3.001)	<0.001	2.014 (1.474, 2.753)	<0.001	1.824 (1.333, 2.496)	<0.001	1.672 (1.218, 2.295)	0.010
G3	3.695 (2.754, 4.957)	<0.001	3.343 (2.488, 4.492)	<0.001	3.024 (2.247, 4.069)	<0.001	2.189 (1.611, 2.973)	<0.001
G4	5.656 (4.243, 7.541)	<0.001	5.202 (3.898, 6.974)	<0.001	4.724 (3.528, 6.326)	<0.001	2.529 (1.769, 3.614)	<0.001
HFpEF								
G1	1		1		1		1	
G2	2.244 (1.339, 3.763)	0.020	2.042 (1.217, 3.428)	0.007	1.924 (1.145, 3.235)	0.014	1.950 (1.157, 3.286)	0.020
G3	3.820 (2.317, 6.300)	<0.001	3.207 (1.939, 5.303)	<0.001	2.867 (1.728, 4.757)	<0.001	2.511 (1.505, 4.189)	0.004
G4	5.587 (3.434, 9.091)	<0.001	4.919 (3.018, 8.011)	<0.001	4.208 (2.563, 6.911)	<0.001	3.087 (1.850, 5.148)	0.022
HFrEF + HFmrEF								
G1	1		1		1		1	
G2	2.186 (1.479, 3.321)	<0.001	2.004 (1.353, 2.968)	0.001	1.718 (1.200, 2.643)	0.004	1.693 (1.133, 2.530)	0.015
G3	3.625 (2.521, 5.213)	<0.001	3.343 (2.320, 4.817)	<0.001	3.048 (2.112, 4.399)	<0.001	2.664 (1.826, 3.885)	<0.001
G4	5.710 (3.997, 8.159)	<0.001	5.322 (3.726, 7.629)	<0.001	5.137 (3.430, 7.065)	<0.001	3.827 (2.624, 5.580)	<0.001

Group 2, Group 3 and Group 4 all used Group 1 as the reference group. Model 1: adjust for age; Model 2: adjust for age, NHYA cardiac function class and heart rate; Model 3: adjust for Model 2 + IgBNP, uric acid, chlorine, hemoglobin, total cholesterol and creatinine. HR, hazard ratio; CI, confidence interval; CAR, c-reactive protein-to-albumin ratio; HFpEF, heart failure with preserved ejection fraction; HFrEF, heart failure with reduced ejection fraction; HFmrEF, heart failure with mildly reduced ejection fraction. G1: CAR ≤7.67, G2: 7.67 < CAR ≤ 20.95, G3: 20.95 < CAR ≤ 63.27, G4: CAR >63.27.

### 3.4 Ability of CAR to Predict All-Cause Mortality in HF Patients 

We constructed separate ROC curves for CAR and CRP in patients with different ejection fractions to evaluate the area under the curve (AUC), and the sensitivity and specificity of the CAR for predicting all-cause mortality were obtained. In all the HF patients, the AUC for the CAR was 0.732 (95% CI = 0.704–0.760, *p* < 0.001), the sensitivity and specificity were 66.2% and 72.7%, respectively, and the AUC for CRP was 0.729 (95% CI = 0.701–0.757, *p* < 0.001). In HFpEF patients, the AUC for the CAR was 0.727 (95% CI = 0.681–0.773, *p* < 0.001), the sensitivity and specificity were 76% and 59.5%, and the AUC for CRP was 0.724 (95% CI = 0.679–0.770, *p* < 0.001). In HErEF plus HEmrEF HF patients, the AUC for the CAR was 0.737 (95% CI = 0.701–0.773, *p* < 0.001; sensitivity and specificity = 66.1% and 71%, respectively; and the AUC for CRP was 0.733 (95% CI = 0.697–0.769, *p* < 0.001). Statistical results of the ROC curve showed that CAR was superior to CRP alone in predicting all-cause death (Fig. 3A, Fig. 3B Fig. 3C).

**Fig. 3A.**
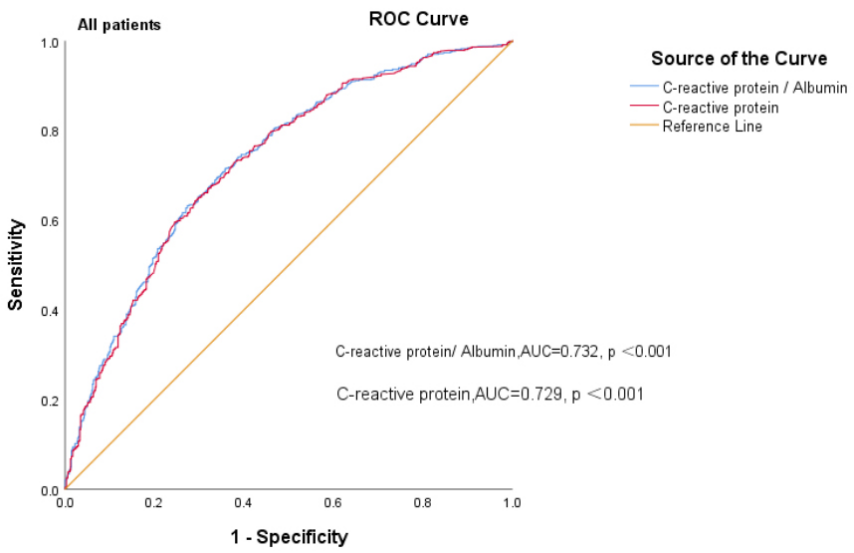
**Receiver operating curves of CAR levels for predicting all CHF patients’ mortality**. AUC, area under curve; ROC, receiver operating characteristic; CAR, c-reactive protein-to-albumin ratio; CHF, chronic heart failure.

**Fig. 3B.**
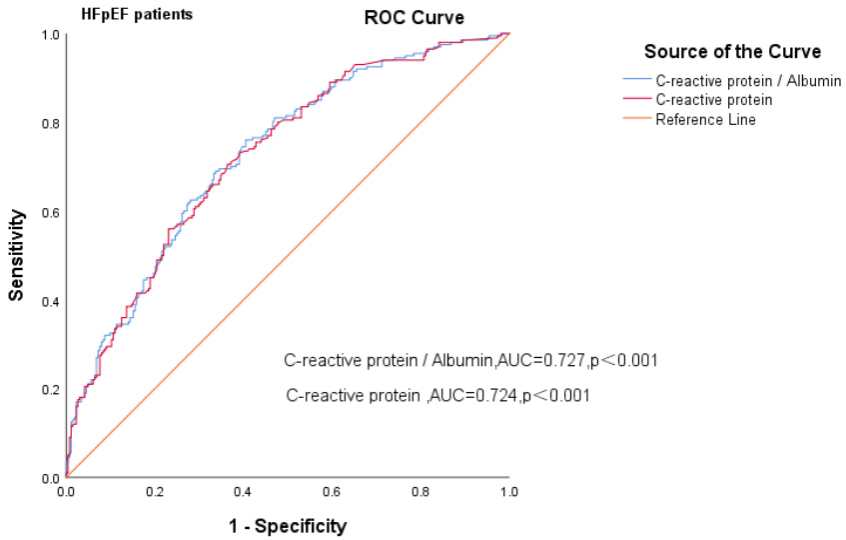
**Receiver operating curves of CAR levels for predicting HFpEF patients’ mortality**. AUC, area under curve; HF, heart failure; HFpEF, heart failure with preserved ejection fraction; ROC, receiver operating characteristic; CAR, c-reactive protein-to-albumin ratio.

**Fig. 3C.**
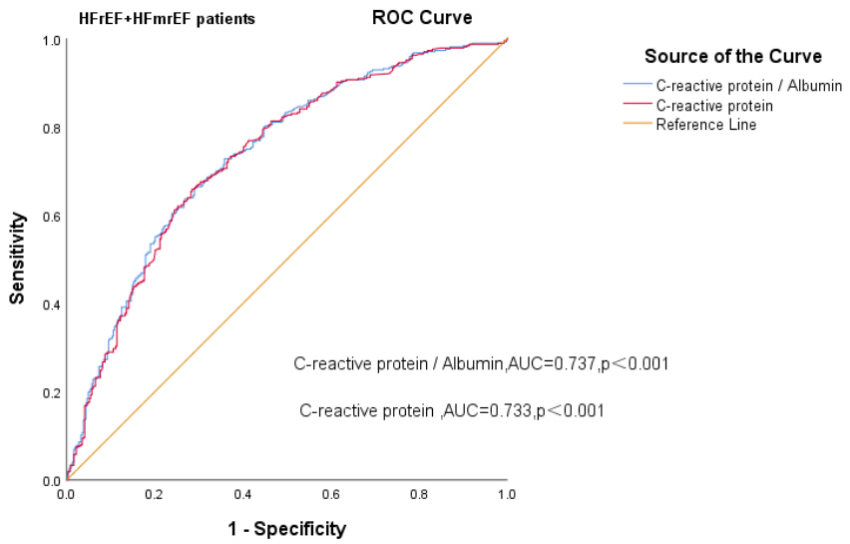
**Receiver operating curves of CAR levels for predicting HFrEF plus HFmrEF patients’ mortality**. AUC, area under curve; HFrEF, heart failure with reduced ejection fraction; HFmrEF, heart failure with mildly reduced ejection fraction; ROC, receiver operating characteristic; CAR, c-reactive protein-to-albumin ratio.

## 4. Discussion

This was a retrospective study, and we evaluated the relationship between the CAR and the clinical features of patients with HF. Our findings indicated that a higher CAR was associated with a significantly increased risk of all-cause mortality in HF patients with different ejection fractions. The CAR was an independent predictor of death regardless of the CHF subtype (HFpEF or HFrEF + HFmrEF).

The results of the statistical analysis demonstrated that the impact of the CAR on the prognosis of patients with heart failure was significant. The Kaplan-Meier analysis revealed that all-cause mortality was always highest in Group 4 (CAR >63.27) and lowest in Group 1 (CAR ≤7.67), both in patients with HFpEF and in patients with HFrEF plus HFmrEF. By Cox proportional hazards analysis, we identified CAR levels as an independent predictor of unfavourable prognosis and all-cause mortality in all HF patients and in different HF subtypes (HFpEF and HFrEF plus HFmrEF). According to hazard ratios for mortality in different subtypes of heart failure, Group 4 always exhibited the highest risk of death when Group 1 was used as a reference. The statistical findings clearly indicate that the risk of all-cause mortality in patients with heart failure was at its highest when the concentration of CAR was >63.27. The ROC curves revealed that the AUC for the CAR was 0.732 (*p* < 0.001) for all HF patients, 0.727 for HFpEF patients and 0.737 for HFrEF plus HFmrEF patients (*p* < 0.001). The CAR has good predictive value for the prognosis of HF patients.

CRP is a protein synthesized by the liver to identify the presence of inflammation in the body. Infection and tissue injury, such as acute myocardial infarction, surgical trauma, tumours and other factors, can also cause CRP levels to increase [11]. Several recent studies have demonstrated that the concentration of CRP is a strong predictor of the occurrence or exacerbation of heart failure events in both patients with heart failure and high-risk populations [12]. CRP can be considered a predictor of all-cause mortality risk in patients with CHF [14].

Albumin is one of the main proteins present in human blood plasma and is synthesized by the liver. Heart failure patients often experience a loss of appetite, dyspepsia and other conditions, resulting in insufficient nutrient intake and reduced albumin synthesis [19]. Patients with heart failure often have a chronic inflammatory response that may lead to impaired liver function, resulting in reduced albumin synthesis. Moreover, inflammation also increases the permeability of capillaries, leading to leakage of plasma ALB, which leads to a decrease in plasma ALB levels [16, 27]. As heart failure progresses, people with heart failure often have other chronic conditions, such as chronic kidney disease and liver disease, that may result in reduced albumin synthesis or increased loss [18].

The CAR is a comprehensive indicator of inflammation and nutritional status. The CAR was first evaluated in patients with cancer, surgical patients and critical illnesses and was found to have better predictive value than CRP or ALB alone for adverse outcomes [20, 21, 22, 23, 24, 25, 26, 28]. Recently, Lele Cheng *et al*. [17] reported that in patients with chronic total coronary occlusion (CTO) who underwent percutaneous coronary intervention (PCI), the risk of all-cause death, cardiovascular death, and major adverse cardiac events (MACEs) increased with increasing CAR in patients with CTO. Ozkan *et al*. [29] studied the ratio of CRP to albumin in cryoablation patients and reported that the CAR was a better predictor of atrial fibrillation recurrence than CRP or ALB alone. Jiawen Li *et al*. [30] demonstrated that a higher CAR was associated with worse 5-year outcomes among diabetic patients with PCI.

In cases of severe heart failure, inflammation and difficulty absorbing nutrients result in significant changes in both CRP levels and serum albumin concentrations. The research showed that patients within the highest quartile for CAR had a higher likelihood of mortality compared to those in the other quartile, as revealed in both unadjusted and adjusted analyses. Moreover, the use of CAR can help assess the level of risk in people with heart failure, leading to enhanced surveillance and improved treatment outcomes. Both the CRP and ALB levels are routine laboratory test results that can be controlled with a variety of clinical treatments. The various pathophysiological changes observed in heart failure involve the expression of related biomarkers, and these biomarkers play important roles in predicting, diagnosing, and guiding treatment and evaluating the prognosis of heart failure. Monitoring biomarker levels in patients with heart failure is an important part of heart failure management. The results of this study may help clinicians implement early interventions, such as aggressive anti-inflammatory therapy and malnutrition correction, to reduce mortality in patients with heart failure. This finding has rarely been mentioned in studies related to heart failure.

The study currently has the following limitations. Firstly, this is a single-center retrospective study, and issues such as selection bias and missing data need to be further addressed, or more multi-center data should be included for research. Secondly, the CAR data collected were mostly test indicators of the time of admission, and the prognostic impact of CAR changes before and after treatment should also be considered. Thirdly, our primary study included patients with NYHA Class III or IV disease. Therefore, some of the findings may not apply to patients with relatively mild symptoms of heart failure. Finally, our limited sample size makes it challenging to account for all potentially confounding factors. In the future, the data will be refined to improve the research.

## 5. Conclusions

Our study reveals that CAR is a potential independent predictor of prognosis in patients with heart failure. CAR has a significant effect on the prognosis of patients with heart failure and can be used as an effective predictor of patient prognosis. When the CAR was >63.27, HF patients had the highest risk of all-cause death. Both the CRP and ALB levels are readily available test results that can be influenced by a variety of clinical treatments. Through the indicator of CAR, doctors can identify potential patients with chronic heart failure exacerbations in clinical work, and carry out early intervention and treatment strategies for risk factors, so as to reduce the incidence of chronic heart failure exacerbation, delay disease progression, and reduce the mortality of chronic heart failure patients.

## Availability of Data and Materials

The datasets during and/or analysed during the current study available from the corresponding author on reasonable request.
